# A comparative study of diagnostic accuracy in 3026 pleural biopsies and matched pleural effusion cytology with clinical correlation

**DOI:** 10.1002/cam4.5038

**Published:** 2022-07-18

**Authors:** Ivan K. Poon, Ronald C. K. Chan, Joseph S. H. Choi, Joanna K. M. Ng, Katsie T. Tang, Yolanda Y. H. Wong, Ka Pang Chan, Wing Ho Yip, Gary M. Tse, Joshua J. X. Li

**Affiliations:** ^1^ Department of Anatomical and Cellular Pathology, Prince of Wales Hospital The Chinese University of Hong Kong Shatin Hong Kong; ^2^ Department of Medicine and Therapeutics, Prince of Wales Hospital The Chinese University of Hong Kong Shatin Hong Kong

**Keywords:** cytopathology, lung cancer, metastasis, risk assessment

## Abstract

**Background:**

Pleural effusion can be caused by a wide range of benign and malignant conditions. Pleural biopsy and effusion cytology represent two key methods of pathological diagnosis. To compare the performance these two methods, a large cohort of matched pleural biopsy and effusion cytology with clinical follow‐up was reviewed.

**Methods:**

Pleural biopsies and effusion cytology specimens over a period of 18 years were retrieved. Cytology specimens collected within 7 days of pleural biopsy were matched. Reports were reviewed, and the cause for pleural effusion was determined by hospital disease coding and clinical data.

**Results:**

Totally, 3026 cases were included. The leading cause of benign effusion was tuberculosis (*n* = 650). Malignant pleural effusion (MPE) was more common in older females (*p* < 0.001) and mostly due to lung cancer (*n* = 959), breast cancer (*n* = 64), and mesothelioma (*n* = 48). The inadequate/insufficient (B1/C1) rate of biopsy was higher than cytology (15.6% vs. 0.3%) but the rates for other diagnostic categories were similar. Biopsy and cytology showed a correlation coefficient of 0.315, improving to 0.449 when inadequate/insufficient (B1/C1) cases were excluded. The ROM for benign cytology (C2) was lower than biopsy (B2) (*p* < 0.001). Compared with biopsy, the diagnostic accuracy was higher in cytology overall and for metastatic carcinomas (*p* < 0.001) but lower for hematolymphoid malignancies (*p* = 0.014) and mesotheliomas (*p* = 0.002).

**Conclusions:**

These results suggest that effusion cytology may be better for confirming benignity and diagnosing carcinomatous MPE. In these cases, pleural biopsy may be withheld to reduce procedural risks. However, for suspected hematolymphoid malignancies and mesothelioma, biopsy should be considered.

## Graphical abstract

Effusion cytology is superior to pleural biopsy in excluding malignant pleural effusion and diagnosis of metastatic carcinomas.

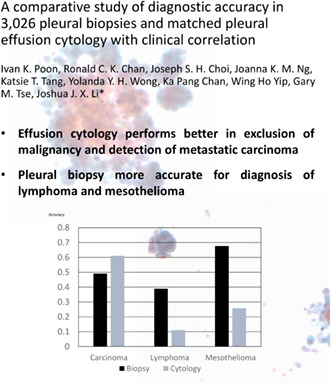

## 1 INTRODUCTION

Pleural effusion is frequently encountered in hospital admissions. The underlying causes include a wide range of benign, reactive, infective, autoimmune, and neoplastic diseases.^1^ Tissue diagnosis is crucial for the diagnosis and subsequent management of malignant pleural effusion (MPE), requiring either pleural fluid cytology or pleural biopsy.^2^, ^3^ While thoracentesis for pleural fluid cytology is minimally invasive and cost‐efficient, tissue biopsy is generally considered more definitive and targeted for obtaining a larger volume of tissue.^4^ However, in the context of pleural effusion, fluid cytology has the potential advantage of sampling exfoliated cells from the entire pleural cavity.^5^ The diagnostic accuracy and yield of these two methods may vary according to the nature of pleural effusion, as small or mediastinal pleural lesions can be difficult to biopsy,^6^ whereas tumor cells shed to pleural fluid may be scanty in sarcomas.^7^ A direct comparison of the diagnostic accuracy and yield of these two methods, with respect to the underlying condition, is necessary for selecting the optimal mode of tissue acquisition. In this study, we reviewed a large cohort of matched pleural effusion cytology and biopsy, compared the diagnostic concordance of these two methods, and further analyzed their accuracy by reviewing hospital disease coding confirmed with clinical, radiological, microbiological, serological, and /or pathological data.

## 2 METHODOLOGY

**FIGURE 1:**
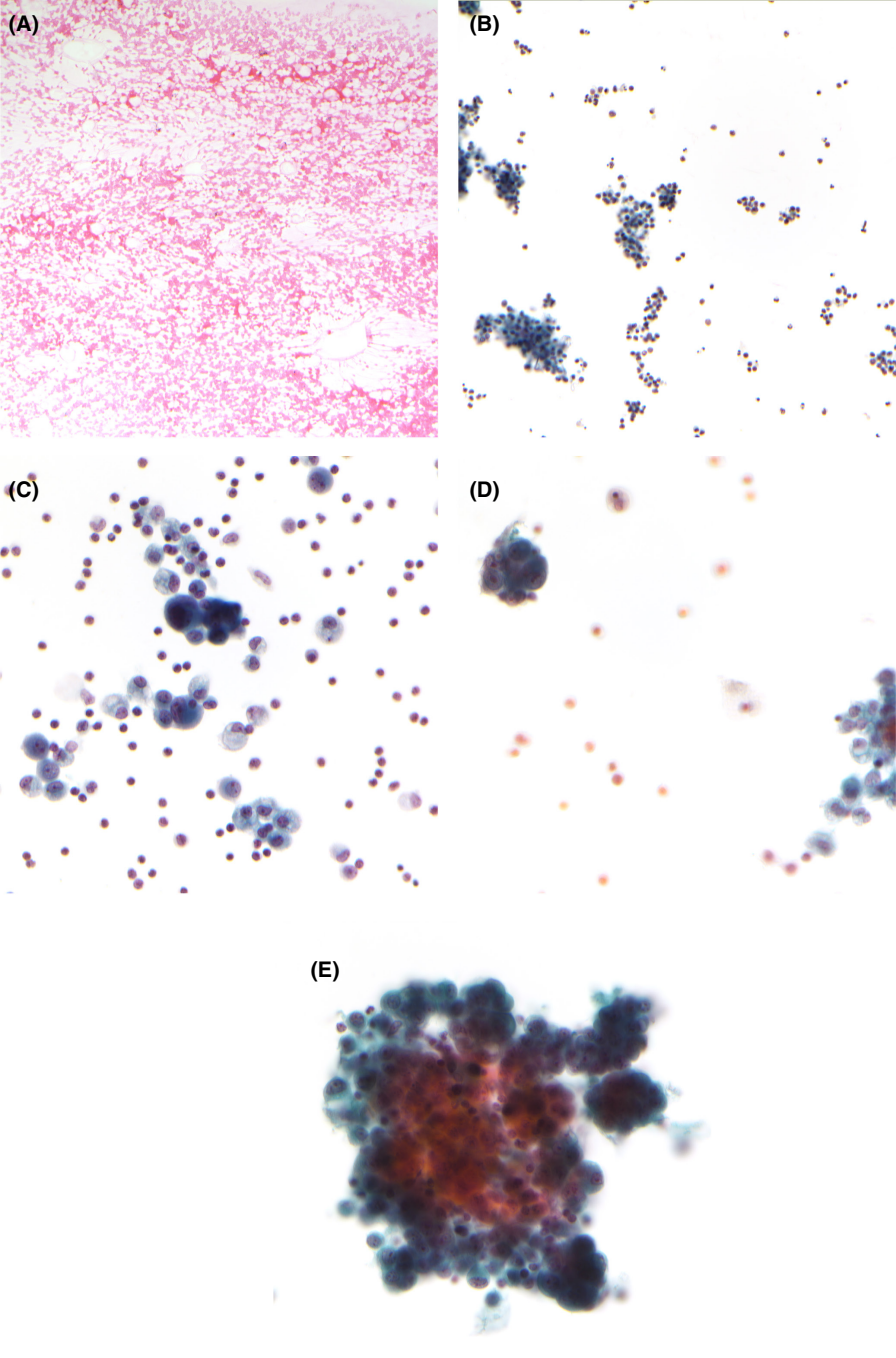
Examples of cytology specimens with (A) C1, (B) C2, (C) C3, (D) C4, and (E) C5 diagnoses. (A) C1—only blood without mesothelial cells, follow‐up was pleural lipoma (smear, 10x, H&E); (B) C2—scattered mesothelial cells among background inflammatory cells, follow‐up was pneumonia (cytospin, 20x, pap stain); (C) C3—atypical cells with hyperchromatic but smudged nuclei, follow‐up was metastatic adenocarcinoma of lung (cytospin, 40x, pap stain); (D) C4—rare atypical cells with prominent nucleoli and enlarged nuclei, follow‐up mesothelioma (cytospin, 40x, pap stain); and (E) C5—malignant cells forming cohesive three‐dimensional clusters, follow‐up was metastatic adenocarcinoma of lung (cytospin, 40x, pap stain).

A computerized search was performed in the department archives for pleural biopsies and effusion cytology specimens collected from February 2001 to February 2019. The reports were reviewed for patient demographical data and histologic/cytologic diagnosis. The diagnoses were classified into five‐tiered categories (B1‐5/C1‐5—1: insufficient/inadequate, 2: benign, 3: atypia, 4: suspicious, and 5: malignant; B‐biopsy, C‐cytologic aspirate) (Figure 1), and the microscopic description and diagnostic line of the reports were reviewed for diagnostic classifiers, such as lymphocytosis in effusion cytology or line of differentiation in neoplastic pleural biopsies. To establish the underlying cause of pleural effusion, hospital disease coding and relevant clinical data were retrieved. For malignant conditions, a corresponding hospital disease coding was acquired and in addition confirmed by clinical case notes, radiology reports, tumor markers, and/or previous pathological diagnoses; for benign causes, either an attributable clinical cause has to be documented (e.g., infection, renal failure) or a period of at least 1 year of unremarkable follow‐up has to be recorded.

Pleural biopsies were paired with effusion cytology specimens by a hospital accession number unique to each patient in the region‐wide clinical management system. Only effusion cytology specimens collected within 7 days before or after the date of pleural biopsy were considered a match. When multiple cytology specimens were matched, the most high‐grade diagnosis was taken as the final diagnosis. This study was approved by the Joint Chinese University of Hong Kong—New Territories East Cluster Clinical Research Ethics Committee with an exemption of requirement of written consent (reference number: 2020.289).

Statistical analysis was performed with the statistics software R 4.10. Descriptive statistics and predictive values—sensitivity (Sn), specificity (Sp), positive predictive value (PPV), negative predictive value (NPV), accuracy, and risk of malignancy (ROM) were calculated. The *t* test and Chi‐square test were used for comparison of continuous and categorical variables. κ statistic was used to measure the concordance between pleural biopsy and effusion cytology diagnoses. A *p* value of <0.05 was considered statistically significant.

## 3 RESULTS

### 3.1 Demographics

A total of 3026 pleural biopsies with 6194 paired pleural effusion cytology specimens were retrieved. These were derived from 2460 patients (1466 and 994 male and female patients, respectively), with an average age of 65.6 years. Each pleural biopsy was on average matched with approximately three cytology specimens, and there were 245 cytology specimens matched to more than one pleural biopsy (Table 1). There were 1686 cases of benign and 1340 malignant effusions. The common causes of benign effusion were tuberculosis (*n* = 650/1686, 38.6%), parapneumonic effusion (*n* = 524/1686, 31.1%), and heart failure (*n* = 184/1686, 10.9%). Metastatic lung cancer was the leading cause of MPE (*n* = 959/1340, 71.6%), followed by metastatic breast cancer (*n* = 64/1340, 4.8%) and mesothelioma (*n* = 48/1340, 3.6%) (Table 2). The mean patients' age in benign cases (63.5 years) was significantly lower than malignant cases (68.6 years) (*p* < 0.001). Effusions due to hematolymphoid malignancies were observed in the youngest age group (average 55.6 years) compared with metastatic carcinomas which occurred in the oldest age group (average 69.6 years). MPEs were more common in older (*p* < 0.001) and female (*p* < 0.001) patients. There was a preponderance of male patients for mesothelioma (42/48, 87.5%, *p* < 0.001) (Table 1).

**TABLE 1:** Demographics of the cohort

No. of patients	2460
Age (mean, range)	65.6 (1–101)
Sex (M/F)	1466/994
No. of pleural biopsies	3026
No. of effusion cytologies	6194
No. of cytology specimens per pair (mean, range)	3.1 (1–14)

	Benign	Malignant			
		Total	Carcinoma	Mesothelioma	Hematolymphoid malignancy
Average age	63.5	68.6	69.6	68.0	55.6
*p* value	<0.001	<0.001	<0.001	0.203	<0.001
Sex (M/F)					
Male	1147	684	596	42	31
Female	539	656	616	6	20
*p* value	<0.001	<0.001	<0.001	<0.001	0.757

**TABLE 2:** Breakdown of clinical diagnoses of the cases included

Type	Subtype	No.	Total	Type	Subtype	No.	Total
Benign				Malignant			
Reactive/inflammatory	Benign, NOS	30		carcinoma	Breast	64	
	Fluid overload	76			Carcinoma of unknown primary	50	
	Heart failure	184			Female genital tract	27	
	Kidney failure	47			Head and neck	11	
	Liver failure	31			Kidney	15	
	Chylothorax	1			Liver	4	
	Pericarditis	1			Lower gastrointestinal tract	12	
	Interstitial lung disease	1			Lung	959	
	Meig's syndrome	3			Multiple	14	
	Ovarian hyperstimulation syndrome	2			Nasopharynx	10	
	Pleuroperitoneal fistula	4	380		Pancreaticobiliary	8	
					Primary peritoneal	2	
Autoimmune	Autoimmune, NOS	2			Prostate	6	
	Systemic Lupus Erythematosus	20			Skin	1	
	Dermatomyositis	1			Thymus	9	
	Rheumatoid arthritis	1			Thyroid	2	
	Still's disease	2			Upper gastrointestinal tract	18	1212
	Vasculitis	1	27				
				Hematolymphoid malignancy	Acute lymphoblastic leukemia	1	
Infection	Abscess	4			Acute lymphocytic leukemia	1	
	Atypical mycobacterium	3			Acute myeloid leukemia	2	
	AECOPD	20			Anaplastic large cell lymphoma	2	
	Bronchiectasis	1			B‐cell lymphoma, NOS	4	
	Empyema	66			CLL/SLL	1	
	Fungus	2			Chronic myeloid leukemia	1	
	Mediastinitis/pleuritis	3			Diffuse large B‐cell lymphoma	13	
	Pneumonia	524			Extranodal marginal zone lymphoma	1	
	PTB/granulomatous inflammation	650	1273		Follicular lymphoma	4	
					Hodgkin lymphoma	1	
Other neoplasms	Amyloid	2			Lymphoma, NOS	5	
	Solitary fibrous tumor	1			Lymphoplasmacytic lymphoma	4	
	Leiomyoma	1			Mantle cell lymphoma	1	
	Lipoma	1			Myeloma/plasma cell neoplasm	3	
	Benign nerve sheath tumor	1	6		Primary effusion lymphoma	1	
					T‐lymphoblastic lymphoma	6	51
Malignant							
mesothelioma		48	48	Sarcoma/melanoma	Angiosarcoma	2	
					Ewing's sarcoma	1	
Other neoplasms	Thymoma	2			Leiomyosarcoma	1	
	Atypical carcinoid	1	3		Melanoma	3	
					Malignant fibrous histiocytoma	2	
					Malignant peripheral nerve sheath tumor	2	
					Osteosarcoma	9	
					Sarcoma, NOS	6	26
					Grand Total		3026

Abbreviations: AECOPD, acute exacerbation of chronic obstructive pulmonary disease; CLL/SLL, chronic lymphocytic leukemia/small lymphocytic lymphoma; NOS, not otherwise specified; PTB, pulmonary tuberculosis.

### 3.2 Distribution and concordance of diagnoses

For pleural biopsy, there were 473 (15.6%) B1, 1890 (62.5%) B2, 100 (3.3%) B3, 50 (1.7%) B4, and 513 (17.0%) B5 diagnoses, respectively. The biopsies were paired to 9 (0.3%) C1, 2029 (67.1%) C2, 247 (8.2%) C3, 175 (5.8%) C4, and 566 (18.7%) C5 cytologic diagnoses, respectively, from a total of 22 (0.4%) C1, 4159 (67.1%) C2, 471 (7.6%) C3, 344 (5.6%) C4, and 1198 (19.3%) C5 effusion cytology specimens (Table 3). Detailed breakdowns of subclassifications in pleural biopsy and effusion cytology are listed in Table 3.

**TABLE 3:** Breakdown of pathological diagnosis of a) pleural biopsies, b) pleural effusion cytology specimens, and c) paired cytology specimens

a.				b.				c.
Diagnosis		No.	Total	Diagnosis		No.	Total	
B1	NS	473	473	C1	NS	22	22	C1
B2	NS	952		C2	NS	3585		C2
	Inflammation	531			Inflammation	240		
	Granuloma	405			Lymphocytosis	334	4159	
	Mesothelial	1						
	Other neoplasms	1	1890					
B3	NS	82		C3	NS	436		C3
	Mesothelial	14			Lymphoid	26		
	Lymphoid	2			Mesothelial	8		
	Other neoplasms	2	100		Squamous	1	471	
B4	NS	29		C4	NS	333		C4
	Carcinomatous	19			Lymphoid	7		
	Mesothelial	2	50		Mesothelial	4	344	
B5	NS	1		C5	NS	1188		C5
	Carcinomatous	464			Lymphoid	6		
	Mesothelial	20			Mesothelial	4	1198	
	Lymphoid	14						
	Sarcoma	13						
	Other neoplasms	1	513					
	Grand Total:		3026		Grand Total:		6194	

Abbreviation: NS, not specified.

The diagnoses of biopsy and cytology showed an overall concordance of 0.626 with a κ statistic of 0.315, which improved to a concordance of 0.743 and a κ statistic of 0.449 when all B1 and/or C1 cases were excluded.

### 3.3 ROM

The ROM of the B1/2/3/4/5 categories were 0.362, 0.276, 0.900, 0.960, and 0.994, respectively, whereas the ROM of the C1/2/3/4/5 categories were 0.333, 0.209, 0.712, 0.983, and 0.996, respectively. The ROMs of the B2/C2 and B3/C3 categories were significantly higher in biopsy than cytology (*p* < 0.001). Considering B2/C2 diagnoses as negative and B5/C5 as positive, the Sp, Sn, PPVs, NPVs, and accuracies were 0.998, 0.495, 0.994, 0.742, and 0.782 for biopsy, and 0.998, 0.570, 0.996, 0.791, and 0.835 for cytology. Alternatively, when B4/C4 diagnoses were also considered positive, the Sp, Sn, PPVs, NPVs, and accuracies were 0.996, 0.517, 0.991, 0.724, and 0.786 for biopsy and 0.997, 0.634, 0.993, 0.791, and 0.845 for cytology. Both conditions resulted in a significant difference in accuracy (*p* < 0.001) favoring cytology (Table 5).

For subgroup analyses, when B2 and C2 were defined as negative and B5 and C5 were defined as positive, the accuracy of the metastatic carcinoma group was higher for cytology (0.611 vs. 0.491, *p* < 0.001) but lower in the mesothelioma (0.258 vs. 0.677, *p* = 0.002) and hematolymphoid malignancy (0.111 vs. 0.389, *p* = 0.014) groups compared with biopsy. Adding B4 and C4 as positive test results showed similar results (Table 6). The ROMs and specific ROMs stratified according to diagnostic classifiers are listed in Table 7.

## 4 DISCUSSION

In this cohort of 3026 paired pleural biopsies and effusion cytology specimens, there were more male than female patients (*p* < 0.001) and benign than malignant diagnoses (*p* < 0.001). These demographic differences were also reported in cohorts of pleural effusion without paired biopsy and cytology,^8^, ^9^, ^10^ and may be contributed delayed health‐seeking behaviors and higher smoking prevalence in the local male population.^11^, ^12^ The leading cause of benign effusion was tuberculosis, corresponding to the high incidence in the locality^13^ and similar to tuberculosis endemic areas.^14^, ^15^ It was followed by parapneumonic effusion and heart failure. Other causes included autoimmune, infective, and inflammatory reactive conditions and organ failure (Table 2). For MPEs, the common underlying malignancies were lung cancer, breast cancer, and mesothelioma. Lung and breast cancers are known for their high propensity of developing pleural metastasis and MPE,^16^, ^17^ and the majority of patients with mesothelioma suffer from pleural effusion on presentation.^18^ In line with the literature, MPEs more commonly presented in older and female patients than benign effusions (*p* < 0.001),^8^, ^10^ and mesotheliomas were predominantly observed in male patients (*p* < 0.001).^19^ The female genital tract (2.2%, *n* = 27/1212) followed the lung (79.1%, *n* = 959/1212) and the breast (4.1%, *n* = 50/1212) as the third most common primary site contributing to MPEs caused by metastatic carcinomas. The distribution of primary tumors was similar to previous studies, including a cohort of Asian demographic.^15^, ^20^, ^21^ Despite that metastatic carcinoma of lung primary was more common in male patients (52.2%, *n* = 501/959), breast and gynecological cancers in the cohort resulted in an overall predisposition to female patients for MPEs caused by metastatic carcinoma (50.8%, *n* = 616/1212) (Table 1).

There was a higher rate of inadequacy/insufficiency in pleural biopsy compared with effusion cytology (15.6% vs. 0.3%). As the cohort was collected prior to the publication of International System for serous fluid cytopathology, fluid volume was not considered for the diagnosis of insufficiency. Specimens were considered inadequate/insufficient when no mesothelial or lesional cells were included. The rate of inadequacy for pleural biopsy reported in the literature was variable, ranging from 7% to 32%,^22^, ^23^, ^24^, ^25^ and the rate was influenced by the techniques applied (e.g., width of needle gauge, use of image guidance, level of operator experience).^25^ For effusion cytology, the current insufficiency rate was about 1% but higher rates of up to 6% have been reported.^24^, ^26^, ^27^ Excluding the B1/C1 categories, the percentages of other diagnostic categories were similar between pleural biopsy and effusion cytology. The concordance between biopsy and cytology improved when B1 and/or C1 cases were excluded. However, it should be noted that there were 27 C4 diagnoses and 47 C5 diagnoses matched to B1 pleural biopsies. Out of these 74 pairs, 72 had an underlying malignant condition. On the contrary, only two B5 and no B4 biopsies were matched to C1 diagnoses, and only one (*n* = 1/2, 50%) was malignant on the follow‐up (Table 4).

**TABLE 4:** Concordance between diagnoses of pleural biopsy and paired effusion cytology

Cytologic diagnosis	Biopsy diagnosis					Total
	B1	B2	B3	B4	B5	
C1	3	4	0	0	2	9
C2	349	1548	34	5	93	2029
C3	47	135	19	4	42	247
C4	27	64	11	11	62	175
C5	47	139	36	30	314	566
Total:	473	1890	100	50	513	3026
	Overall concordance					0.626
	κ					0.315
		
	Concordance excluding B1/C1 specimens					0.743
	κ					0.449

Comparing the ROMs of each diagnostic category between biopsy and cytology, only the B2/C2 and B3/C3 categories showed significant difference. The lower ROM of the C3 in cytology may stem from the diagnostic uncertainty in cytology specimens,^28^ contributed by the interpretative difficulties and qualitative issues such as degeneration and preparation artifacts.^29^ Although cytology appears to show superior diagnostic accuracy in the B2/C2 category and all categories combined (Table 5), in subgroup analysis, the difference is only reproduced in cases of metastatic carcinoma (Table 6). Biopsy outperforms cytology for diagnosing hematolymphoid malignancies (*p* = 0.014) and mesothelioma (*p* = 0.002) (Table 6). The majority of MPEs in this cohort were due to metastatic carcinomas, and this would increase the accuracies in the combined and B2/C2 categories for cytology. Inability of demonstrating invasion and overlapping cytomorphology with reactive mesothelial cells are some of the difficulties in cytologic diagnosis of mesothelioma.^30^, ^31^ As for hematolymphoid malignancies, aspiration disrupts the lymph node architecture and limits the diagnosis of low‐grade lymphomas.^32^ However, in carcinomatous MPEs, previous studies have shown that blinded pleural biopsy only provides limited additional diagnostic information, reflecting the irregular distribution of malignant deposits across the pleura.^33^, ^34^ In contrast, effusion fluid usually immerses the entire pleural cavity, and in this situation effusion cytology may be more representative than pleural biopsy.

**TABLE 5:** Risk of malignancy and predictivity of a) pleural biopsy and b) effusion cytology

a.	Clinical diagnosis				b.	Clinical diagnosis				*p* value
	Benign	Malignant	ROM			Benign	Malignant	ROM		
B1	302	171	0.362		C1	6	3	0.333		1
B2	1369	521	0.276		C2	1604	425	0.209		< 0.001
B3	10	90	0.9		C3	71	176	0.712		< 0.001
B4	2	48	0.96		C4	3	172	0.983		0.6723
B5	3	510	0.994		C5	2	564	0.996		0.9122
B2	1369	521			C2	1604	425			
B5	3	510			C5	2	564			
	Sp	0.998	PPV	0.994		Sp	0.998	PPV	0.996	
	Sn	0.495	NPV	0.742		Sn	0.570	NPV	0.791	
			Accuracy	0.782				Accuracy	0.835	< 0.001
B2	1369	521			C2	1604	425			
B4 + B5	5	558			C4 + C5	5	736			
	Sp	0.996	PPV	0.991		Sp	0.997	PPV	0.993	
	Sn	0.517	NPV	0.724		Sn	0.634	NPV	0.791	
			Accuracy	0.786				Accuracy	0.845	
										< 0.001

Abbreviations: NPV, negative predictive value; PPV, positive predictive value; ROM, risk of malignancy; Sn, sensitivity; Sp, specificity.

**TABLE 6:** Comparison of diagnostic accuracy between pleural biopsy and effusion cytology in clinically confirmed a) hematolymphoid malignancy, b) mesothelioma, and c) metastatic carcinoma

a. Hematolymphoid malignancy					b. Mesothelioma					c. Metastatic carcinoma				
B2	22	C2	32	*p* value	B2	10	C2	23	*p* value	B2	481	C2	351	*p* value
B5	14	C5	4		B5	21	C5	8		B5	464	C5	552	
Accuracy	0.389		0.111	0.014	Accuracy	0.677		0.258	0.002	Accuracy	0.491		0.611	<0.001
B2	22	C2	32		B2	10	C2	23		B2	481	C2	351	
B4 + B5	15	C4 + C5	9		B4 + B5	24	C4 + C5	18		B4 + B5	508	C4 + C5	702	
Accuracy	0.405		0.220	0.126	Accuracy	0.706		0.439	0.037	Accuracy	0.514		0.667	< 0.001

Further review of biopsy and cytology reports of the hematolymphoid malignancy and mesothelioma subgroups from Table 6, immunocytochemistry was performed in 56.8% (*n* = 21/37) and 76.5% (*n* = 26/34) of biopsy cases included for analysis in hematolymphoid malignancy and mesothelioma subgroups. This is higher than the proportion of cytology specimens with immunocytochemistry performed (hematolymphoid malignancy subgroup: 7.3%, *n* = 3/41; mesothelioma subgroup: 4.9%, *n* = 2/41). Immunocytochemistry and other ancillary tests provide additional information for cytologic interpretation, but still have limitations in differentiating reactive lymphoid hyperplasia from low‐grade lymphomas,^35^ and can give contradicting or noncontributory results.^36^ For the diagnosis of mesothelioma, equivocal invasion on solid tissue remains the most decisive diagnostic feature, which outperforms immunocytochemical and serologic markers in terms of specificity and reproducibility.^30^ A lower proportion of cases with immunocytochemistry performed may have contributed to the difference in diagnostic performance between biopsy and cytology. However, barriers to immunocytochemistry exclusive to cytology preparations should also be considered, including insufficient material for conversion to cell block and technical difficulties in antibody staining for alcohol‐fixed specimens.^37^ Cytomorphology remains the crux of cytologic interpretation with ancillary tests adding value only within specific contexts.^38^

For the accuracy in typing of malignant lesions (carcinoma vs. mesothelioma vs. hematolymphoid), analysis was limited by the small number of cases. In addition, a significant number of reports lacked diagnostic classifiers, only issuing a diagnosis of “malignancy” or “malignant cells.” When mesothelial lineage was specified in the report, both cytology and biopsy were very accurate (specific ROMs = 1) (Table 7). The diagnosis of malignant hematolymphoid lesion on biopsy (B5) was correct in 93% of cases (*n* = 13/14), greater than that of cytology (C4: *n* = 4/5, 80%, C5: 3/4, 75%).

**TABLE 7:** Risk of malignancy in a) pleural biopsy and b) effusion cytology subclassified by diagnostic subgroups

Biopsy Dx	Clinical Dx						Cytologic Dx	Clinical Dx					
Lymphoid lesion	Hemato‐lymphoid malignancy	Other malignant lesions	Benign	Total	Specific ROM	Overall ROM	Lymphocytosis/lymphoid	Hemato‐lymphoid malignancy	Other malignant lesions	Benign	Total	Specific ROM	Overall ROM
B2	0	0	0	0	‐	‐	C2	10	26	196	232	0.043	0.155
B3	2	0	0	2	1	1	C3	9	0	3	12	0.75	0.75
B4	0	0	0	0	‐	‐	C4	4	0	1	5	0.8	0.8
B5	13	0	1	14	0.929	0.929	C5	3	0	1	4	0.75	0.75

Mesothelial lesion	Mesothelioma	Other malignant lesions	Benign				Mesothelial lesion	Mesothelioma	Other malignant lesions	Benign			
B2	1	0	0	1	1	1	C2	0	0	0	0	‐	‐
B3	7	5	2	14	0.5	0.857	C3	0	3	2	5	0	0.6
B4	2	0	0	2	1	1	C4	2	0	0	2	1	1
B5	20	0	0	20	1	1	C5	5	0	0	5	1	1

Metastatic carcinoma	Metastatic carcinoma	Other malignant lesions	Benign				NS	Metastatic carcinoma	Other malignant lesions	Benign			
B4	18	0	1	19	0.947	0.947	C4	150	16	2	168	0.893	0.988
B5	462	1	1	464	0.996	0.998	C5	552	4	1	557	0.991	0.998

Abbreviations: Dx, diagnosis; NS, not specified; ROM, risk of malignancy.

Considerations in performing concurrent pleural biopsy at the time of thoracentesis is crucial in the management of pleural effusion. Factors include the availability of resource, likelihood of underlying disease determined by clinical impression and previous workup results, and the added risk of complication for pleural biopsy. For example, bedside blinded pleural biopsy is more commonly performed alongside diagnostic thoracentesis in tuberculosis endemic area for the exclusion of tuberculous pleuritis.^33^, ^39^ In the latest Society of Interventional Radiology consensus guidelines,^40^ pleural biopsy is regarded as deep organ biopsy and should only be performed under strict monitoring of platelet count and clotting profile. Whereas thoracocentesis is considered as a procedure with low bleeding risk, thus does not require withholding anticoagulation or antiplatelet medication beforehand. Therefore, if there is a high pretest probability of carcinomatous MPE, an early diagnostic thoracentesis right after clinical consultation with real‐time ultrasound guidance may be preferable, if the patient has low bleeding tendency or is even on antiplatelet agents.

The techniques in pleural biopsy have undergone significant improvements over the period in which data were collected for this cohort. The performance of pleural biopsy has been improved by prebiopsy ultrasound localization of pleural abnormality and real‐time image guidance.^41^ The sensitivity and accuracy of pleural biopsy in this cohort may have been lowered by the inclusion of blinded biopsy, variation in operator experience, and usage of less recent methods. Another limitation was that the cohort assembled before the introduction of the International system for reporting of serous fluid.^3^ The diagnostic criteria and reproducibility may be negatively impacted. Volume criteria from the International system were not incorporated in this cohort, but its effect may be rather insubstantial as the insufficiency rate was low (0.3%).

## 5 CONCLUSION

This large cohort of paired pleural biopsy and effusion cytology specimens demonstrated that the inadequate/insufficient (B1/C1) rate was higher in biopsy than cytology, while that of the other diagnostic categories were similar. Biopsy and cytology showed fair to moderate concordance depending on whether inadequate/insufficient (B1/C1) specimens were included. The malignant (B5/C5) and suspicious (B4/C4) categories had similar ROMs. The ROM for atypia in biopsy (B3) was higher than that of cytology (C3). Cytology showed better performance in terms of ROM for the benign category (B2/C2) and overall diagnostic accuracy. Subgroup analysis showed that cytology was more accurate in carcinomatous MPEs but not for hematolymphoid malignancies and mesotheliomas. These results suggest that for patients with a high pretest probability of MPE, particularly when metastatic carcinoma is suspected, diagnostic thoracentesis alone may be sufficient as the initial step. However, for workup of hematolymphoid malignancies and mesothelioma, biopsy should be considered.

## ETHICS STATEMENT

This study was approved by the Joint Chinese University of Hong Kong – New Territories East Cluster Clinical Research Ethics Committee (reference number: 2020.289).

## FUNDING INFORMATION

The authors have no funding to declare.

## CONFLICT OF INTEREST

The authors declare that there is no conflict of interest regarding the publication of this paper.

## DECLARATION

The abstract of this manuscript has been accepted for presentation in Pathology Update 2022.

## AUTHOR CONTRIBUTIONS

Ivan K. Poon – conceptualization, data curation, investigation, methodology, formal analysis, and writing—original draftRonald C. K. Chan – conceptualization, methodology, formal analysis, and resourcesJoseph S. H. Choi – software, formal analysis, and visualizationJoanna K. M. Ng – data curation, investigation, and methodologyKatsie T. Tang – data curation and investigationYolanda Y. H. Wong – data curation and investigationKa Pang Chan – validation and writing—original draftWing Ho Yip – validationGary M. Tse – methodology, formal analysis, and writing—review and editingJoshua J. X. Li – conceptualization, data curation, investigation, methodology, formal analysis, and writing—review and editing.

## Data Availability

The data that support the findings of this study are available from the corresponding author, JL, upon reasonable request.
